# CRISPR/Cas9 genome editing for neurodegenerative diseases

**DOI:** 10.17179/excli2023-6155

**Published:** 2023-07-03

**Authors:** Jafar Nouri Nojadeh, Nur Seren Bildiren Eryilmaz, Berrin Imge Ergüder

**Affiliations:** 1 Ankara University Faculty of Medicine, Department of Medical Biochemistry, Ankara, Turkey; 2 The Graduate School of Health Sciences of Ankara University, Ankara, Turkey; 3 Ankara University Faculty of Medicine, Department of Internal Medicine, Ankara, Turkey

**Keywords:** gene editing, neurodegenerative disorders, CRISPR/Cas9, Alzheimer's disease, Parkinson's disease, Huntington's disease, Amyotrophic lateral sclerosis, Spinocerebellar ataxia

## Abstract

Gene therapy has emerged as a promising therapeutic strategy for various conditions, including blood disorders, ocular disease, cancer, and nervous system disorders. The advent of gene editing techniques has facilitated the ability of researchers to specifically target and modify the eukaryotic cell genome, making it a valuable tool for gene therapy. This can be performed through either *in vivo *or* ex vivo* approaches. Gene editing tools, such as zinc finger nucleases, transcription activator-like effector nucleases, and CRISPR-Cas-associated nucleases, can be employed for gene therapy purposes. Among these tools, CRISPR-Cas-based gene editing stands out because of its ability to introduce heritable genome changes by designing short guide RNAs. This review aims to provide an overview of CRISPR-Cas technology and summarizes the latest research on the application of CRISPR/Cas9 genome editing technology for the treatment of the most prevalent neurodegenerative diseases including Alzheimer's disease, Parkinson's disease, Huntington's disease, Amyotrophic lateral sclerosis, and Spinocerebellar ataxia.

## Introduction

Gene therapy is a promising alternative therapeutic strategy for a variety of conditions, including blood disorders, ocular disease, cancer, and nervous system disorders. The advent of gene editing techniques has facilitated the ability of researchers to specifically and selectively target and modify the eukaryotic cell genome, making it a valuable tool for gene therapy (Gonçalves and Paiva, 2017[26]). Genome editing techniques involve breaking the DNA double-strand (DSBs) sequence at specific locations in the genome, which can be repaired using different cellular DNA repair mechanisms, such as endogenous homology-directed repair (HDR), microhomology-mediated end joining (MMEJ), classical non-homologous end joining (cNHEJ), and single-strand annealing (SSA) pathways (Zhao and Hu, 2020[113]). Gene therapy can be performed through either *in vivo *or* ex vivo* approaches (Parambi et al., 2022[74]). The *in vivo* strategy involves delivering genetic material directly to a specific tissue in the patient, while the *ex vivo* strategy involves modifying cells *in vitro* and then transplanting them back into the patient (Gowing et al., 2017[28]; Naldini, 2011[65]). Gene editing tools, including zinc finger nucleases (ZFN), transcription activator-like effector nucleases (TALEN), and clustered regularly interspaced short palindromic repeat (CRISPR)-Cas-associated nucleases, can be employed for gene therapy purposes (Zhao and Hu, 2020[113]). Although each of these gene editing tools has its own advantages, CRISPR-Cas-based gene editing stands out because of its ability to introduce heritable genome changes by designing short guide RNAs for protein-coding genes, rather than using modified proteins, which was one of the main limitations of ZFN and TALEN (Eid and Mahfouz, 2016[21]). The utilization of the CRISPR/Cas9 system for genome editing is a highly effective approach that presents a superior alternative to conventional methods, offering potential advantages for gene therapy (Gopinath et al., 2015[27]). With its capability to delete DNA sequences and correct mutations, CRISPR/Cas9 provides a novel pathway for treating diseases (Ran et al., 2013[80]). This review aims to provide a brief overview of CRISPR-Cas technology, followed by a comprehensive summary of the latest research on the application of CRISPR/Cas9 genome editing technology for the treatment of various neurodegenerative diseases. 

### CRISPR-Cas gene editing system

Clustered Regularly Interspaced Short Palindromic Repeats (CRISPR) were first discovered in Escherichia coli in 1987 (Ishino et al., 1987[34]). Concurrently, several genes that were closely linked to CRISPR and identified as CRISPR-associated (Cas) genes were also identified (Jansen et al., 2002[36]; Makarova and Koonin, 2007[58]). It has been established that CRISPR and RNA-guided Cas proteins work together (Makarova et al., 2006[56]), and the functionality of CRISPR-Cas as an adaptive immune system of prokaryotic cells to defend against invading phages was fully validated by Barrangou's lab in 2007 (Barrangou et al., 2007[4]).

The immune response of CRISPR-Cas has three primary stages: adaptation, expression, and interference (Makarova et al., 2011[57]). During the adaptation phase, a complex of Cas proteins binds to a specific region of DNA, often marked by a short motif known as PAM, and removes a section of the DNA called protospacer. The adaptation complex then inserts the protospacer DNA into the CRISPR array as a spacer, either by duplicating the repeat at the 5' end or by acquiring it from RNA via reverse transcription. In the expression stage, the CRISPR array is transcribed into pre-CRISPR RNA (pre-crRNA) and processed into mature CRISPR RNAs (crRNAs), with each one of them consists of the spacer sequence and certain parts of the flanking repeats. This processing is mediated by different subunits of a Cas complex, a single multidomain Cas protein, or non-Cas host RNases, depending on the CRISPR-Cas variant. During the interference phase, the crRNA remains bound to the processing complex and guides the Cas nuclease to the protospacer or a similar sequence in the genome of a virus or plasmid that is subsequently cleaved and inactivated (Faure et al., 2019[22]; Jiang and Doudna, 2017[38]; Koonin and Makarova, 2019[48]; Makarova et al., 2020[60]; McGinn and Marraffini, 2019[62]). 

The CRISPR-Cas systems can be categorized into two classes based on their effector module design principles. These classes consist of six types (I to VI) and have several subtypes. The Class 1 systems (Type I, III, and IV) contain multi-Cas protein effector complexes, whereas Class 2 systems (Type II, V, and VI) have a single effector protein (Koonin and Makarova, 2019[48]; Makarova et al., 2015[59]). Class 1 CRISPR-Cas systems contain various Cas proteins in their effector module, including Cas3 (sometimes connected to Cas2), Cas5-Cas8, Cas10, and Cas11. The combination of these proteins depends on the system's type and subtype. On the other hand, in class 2 systems, a solitary, significant protein, such as Cas9, Cas12, or Cas13, constitutes the effector module (Makarova et al., 2020[60]).

### CRISPR-Cas9 system

**Figure 1:**
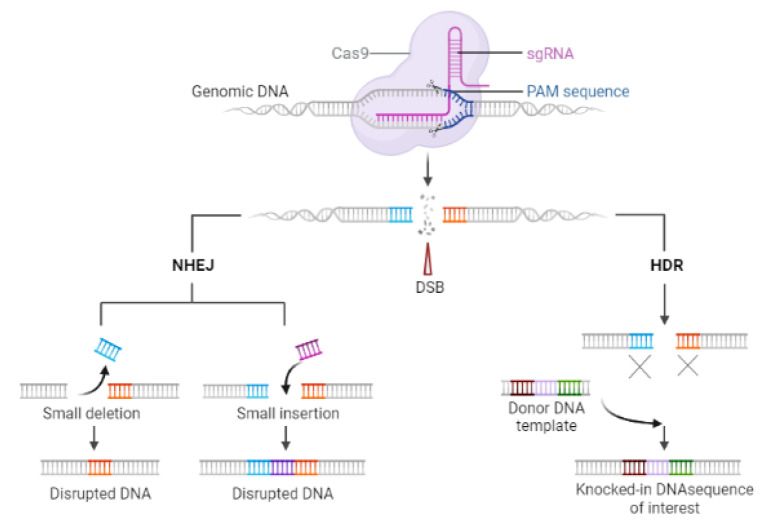
The schematic diagram of CRISPR/Cas9 genome editing illustrates how the Cas9 nuclease forms complexes with sgRNA, enabling the production of specific double-strand breaks (DSBs) in the host DNA. These targeted DNA DSBs can activate the endogenous cellular repair machinery. The DSBs are subsequently repaired through two main mechanisms. Firstly, homology-directed repair (HDR) utilizes the sister chromatid as a template DNA, facilitating precise genomic changes in the host's DNA. Secondly, nonhomologous end joining (NHEJ) occurs when DNA ends are joined without any homology, leading to the introduction of insertions and deletions.

The Type II CRISPR-Cas9 system, which is derived from Streptococcus pyogenes (SpyCas9), is extensively studied and widely employed among various CRISPR-Cas systems for genome modification (Jiang and Doudna, 2017[38]). SpyCas9 is dependent on the presence of two RNA molecules, a crRNA, and a trans-activating crRNA (tracrRNA), to cleave DNA. However, these two RNAs can be combined into a single guide RNA (sgRNA) without altering their individual abilities (Jinek et al., 2012[42]). Briefly, CRISPR-Cas9 system primarily consists of an RNA-guided Cas9 endonuclease and an sgRNA to direct the Cas9 nuclease towards specific location within the genomic DNA sequences (Mali et al., 2013[61]; Ran et al., 2013[80]; Tu et al., 2015[95]). This results in the creation of a double-stranded break (DSB), which can be fixed by two endogenous self-repair mechanisms found within the organism: the non-homologous end joining (NHEJ) pathway or the homology-directed repair (HDR) pathway (Figure 1(Fig. 1)) (Xu and Li, 2020[105]).

**Figure 2:**
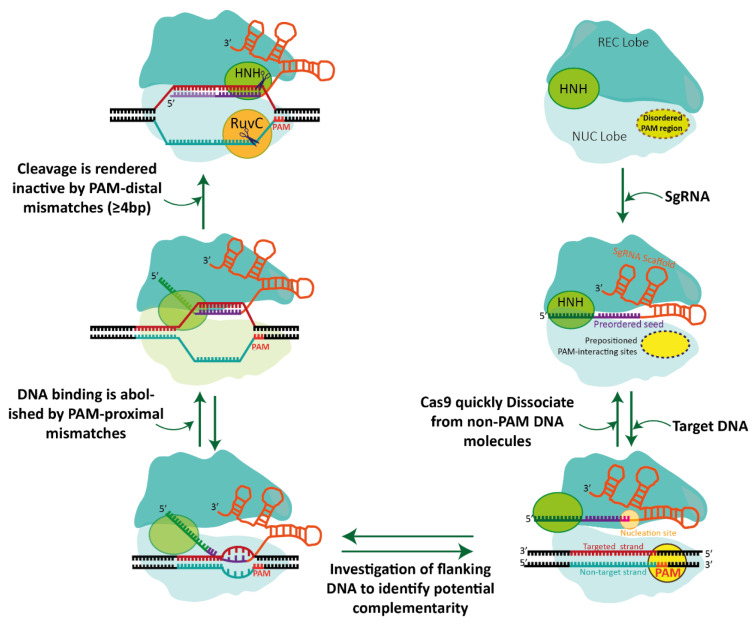
Schematic illustrations demonstrate the proposed mechanisms involved in recognizing and cleaving target DNA using CRISPR-Cas9. When the sgRNA is loaded, Cas9 undergoes a significant rearrangement to enter a target-recognition mode. In this mode, the PAM-interacting cleft (represented by a dotted circle), which is disordered in apo-Cas9, becomes prestructured for PAM sampling. Additionally, the guide RNA seed adopts an A-form helical conformation, facilitating the interrogation of adjacent DNA for guide RNA complementarity. The disordered nonseed RNA nucleotides are represented by dotted white boxes. Further activation of Cas9 occurs through a series of coordinated steps. These steps include PAM recognition, local DNA melting, RNA strand invasion, and stepwise R-loop formation. Concomitantly, the conformational change of the HNH domain leads to allosteric regulation of the RuvC domain, ensuring concerted DNA cleavage. Abbreviations: REC (recognition lobe), NUC (nuclease lobe), PAM (protospacer adjacent motif), bp (base pair), and sgRNA (single-guide RNA) (Jiang and Doudna, 2017)

The Cas9 protein is a complex structure consisting of multiple domains, with two distinct parts referred to as the nuclease (NUC) lobe and the recognition (REC) lobe. These two lobes are linked by a bridge helix (BH) containing a high concentration of arginine amino acids (Anders et al., 2014[2]; Babu et al., 2021[3]; Jiang et al., 2016[39]; Murugan et al., 2017[64]; Yamada et al., 2017[106]; Zhang et al., 2020[112]). The NUC lobe of Cas9 encompasses two endonuclease domains called HNH and RuvC, each of which cuts one strand of the double-stranded DNA being targeted, as well as a PAM-interacting domain to enable the Cas9 protein to bind to the target DNA (Anders et al., 2014[2]; Babu et al., 2021[3]). The Cas9 nuclease and sgRNA form a Cas9 ribonucleoprotein (RNP) capable of binding to and cleaving the specific DNA target (Jinek et al., 2012[41]). In addition, the REC lobe of SpCas9 is composed of several recognition domains (REC1-REC3), which aid in the attachment of SpyCas9 to RNA and DNA (Figure 2(Fig. 2)) (Babu et al., 2021[3]; Jiang and Doudna, 2017[38]; Jiang et al., 2015[40]; Yamada et al., 2017[106]). 

Cas9's reliable and robust function has made it suitable as a programmable tool for generating DNA double-strand breaks both *in vitro *and* in vivo* (Fellmann et al., 2017[23]; Mali et al., 2013[61]; Oakes et al., 2019[68]). Consequently, CRISPR/Cas9 has evolved into an uncomplicated and adaptable RNA-directed system for genome editing that can be used on a variety of organisms and cell types, ranging from bacteria and mice to rats, zebrafish, pigs, human somatic cells, and human pluripotent stem cells for treating human diseases (Cho et al., 2013[14]; Henao-Mejia et al., 2016[33]; Mali et al., 2013[61]; Tu et al., 2015[95]; Vejnar et al., 2016[97]). In 2016, the first instance of CRISPR/Cas9 being used in a clinical setting took place. A group from China administered CRISPR/ Cas9-edited cells to patients with advanced lung cancer as a treatment (Cyranoski, 2016[17]). Over the past few years, numerous clinical trials have been conducted, and several findings have been published. These findings involve the use of CRISPR/Cas9-based treatments for conditions such as acquired immunodeficiency syndrome (AIDS), sickle cell disease (SCD), β-thalassemia, and multiple types of cancers (Guo et al., 2022[29]).

### Delivery system of CRISPR-Cas9

To modify genes in mammalian cells for therapeutic purposes, the CRISPR components need to be delivered into the cells. There are different approaches to deliver the CRISPR/Cas9 components, including viral, mRNA, plasmid, and protein-based methods (Lu et al., 2021[52]). Among these methods, viral delivery is the most commonly used for CRISPR/Cas9. Various virus delivery systems, such as adeno-associated viruses (AAV), Adenoviral Vectors (AdV), and Lentiviral Vectors (LV), have been utilized for this purpose (Xu et al., 2019[104]). AAVs are extensively used in CRISPR genome editing for the following reasons: First, AAVs can penetrate the host cell and persist independently from the host cell genome, allowing the provirus to express continuously and stably for up to 1-2 years, which is advantageous for treating diseases. Second, AAVs can infect a variety of tissues owing to their diverse capsids (Weinmann et al., 2020[101]). Third, AAVs are capable of withstanding changes in pH and temperature while maintaining their stable activity (Rayaprolu et al., 2013[82]).

Another commonly used method for introducing CRISPR technology into cells is by delivering Cas9-encoded mRNA. mRNA-based approaches have a temporary function and avoid the potential risks linked to integrating into the host genome (Nelles et al., 2016[67]). Additionally, these strategies have the advantage of faster results, as mRNA can be transcribed within minutes (Polstein and Gersbach, 2015[78]). 

Plasmid-based approaches are also an attractive method for delivering CRISPR components into cells. This approach offers several benefits. First, gene synthesis is a straightforward process. Second, the created gene can be transferred to the host cell through a plasmid without requiring integration into the host genome, allowing for continuous expression. Additionally, targeted delivery of the CRISPR/Cas9 system to specific organs is crucial for its future applications. Plasmid-based delivery has the potential advantage of enabling tissue or cell-specific targeting by integrating it directly into the plasmid (Lu et al., 2021[52]).

Another effective method for delivering CRISPR components is Protein-based CRISPR/Cas9 strategies, which have revolutionized genetic engineering. In this system, Ribonucleoprotein (RNP) is an essential component of the CRISPR/Cas9 system. In CRISPR-Cas9 RNP delivery system, the Cas9 protein is delivered to the target cells as an RNP complex, which consists of the Cas9 protein and an sgRNA. The RNP complex offers several advantages, including higher specificity, reduced off-target effects, and increased efficiency, making it the attractive method of delivery (Kang et al., 2019[43]; Kim et al., 2014[45]). The Cas9 ribonucleoprotein (RNP) approach is versatile and adaptable to different types of model organisms and cells, including immune cells, primary cells, and stem cells (Schumann et al., 2015[84]; Seki and Rutz, 2018[85]; Wang et al., 2021[100]).

### CRISPR-Cas9 in neurodegenerative diseases

Neurodegeneration refers to the gradual decline of the structure and function of neurons, resulting in the loss of these neural cells. As time goes on, this deterioration progressively worsens and can ultimately lead to nervous system dysfunction (Kovacs, 2019[49]). The group of diseases that are characterized by neurodegeneration are collectively referred to as neurodegenerative diseases (NDs). The most prevalent NDs include Alzheimer's disease, Parkinson's disease, Huntington's disease, Amyotrophic lateral sclerosis, and Spinocerebellar ataxia (Lamptey et al., 2022[50]). The CRISPR-Cas9 system has demonstrated promising results in the treatment of neurodegenerative diseases. In the subsequent sections, we will describe the applications of CRISPR-Cas9 that have been employed for these disorders. 

### Application of CRISPR/Cas9 in Alzheimer's disease

Alzheimer's disease (AD) is a prevalent progressive neurodegenerative disorder that affects millions of people globally, currently ranking as the third leading cause of death following heart disease and cancer (Rohn et al., 2018[83]). The disease is often characterized by memory deficits and cognitive decline, which eventually impacts memory, language, behavior, movement, judgment, and reasoning, leading to dementia and eventual death (Jha et al., 2020[37]; Parambi et al., 2022[74]). AD is mainly characterized by two neuropathological features, the accumulation of extracellular amyloid plaques containing amyloid β-protein (Aβ) and neurofibrillary tangles (NFTs) primarily composed of hyperphosphorylated Tau protein, a microtubule-associated protein. The relationship between these two pathologies is considered the classical hallmark of AD (Šimić et al., 2016[88]; Yan and Vassar, 2014[107]). The classical β-amyloid hypothesis provided a framework for the development of potential disease-modifying therapies that would prevent Aβ formation and promote the elimination of toxic proteins (such as Aβ) from the brain (Rohn et al., 2018[83]). However, despite numerous attempts to develop disease-modifying treatments using animal models of the disease, there have been many failures (Cummings et al., 2014[16]; Galimberti and Scarpini, 2011[25]). Consequently, over the past few years, the CRISPR/Cas9 technology has gained popularity in the field of Alzheimer's disease due to its short experimental duration and low consumption. It is currently being extensively utilized for tasks such as building AD models, identifying pathogenic genes through screening, and for targeted therapy (Lu et al., 2021[52]).

Although the majority of cases of AD are sporadic, a small percentage of cases are familial (known as Familial AD or FAD), caused by dominant autosomal mutations found in one of three genes: amyloid precursor protein (APP), presenilin-1 (PSEN1), and presenilin-2 (PSEN2) (Bertram and Tanzi, 2012[6]; Zhang et al., 2020[111]). PSEN1 mutations are the primary reason behind familial AD and typically result in an earlier onset of symptoms when compared to mutations found in the other two genes. The majority of PSEN1 mutations result in an elevated production of the more aggregation-prone Aβ42 compared to Aβ40 (Shea et al., 2016[86]). This abnormal production of Aβ42 is known to contribute to the formation of Aβ plaques in the brain, which is a hallmark of Alzheimer's disease. Recent studies indicate that the CRISPR/Cas9 technique has the potential to effectively rectify autosomal dominant mutations. This gene editing system has been reported to successfully correct similar types of mutations, further reinforcing its potential for genetic modification. 

A study conducted in 2022 by Konstantinidis et al. suggests that the CRISPR-Cas9 approach can selectively disrupt the PSEN1M146L allele responsible for AD, and partially reverse the abnormal Aβ42/40 ratio that leads to the development of the disease in carriers of this mutation (Konstantinidis et al., 2022[47]). Furthermore, another study has demonstrated that using CRISPR-Cas9 to correct neurons derived from the PSEN2N141I mutated individual fibroblasts can normalize the Aβ42/40 ratio and effectively restore the associated electrophysiological deficits (Ortiz-Virumbrales et al., 2017[70]). Prior research studies that employed CRISPR/Cas9 to correct PSEN gene mutations in FAD by utilizing iPSCs derived from patients provided additional confirmation for these findings (Pires et al., 2016[77]; Poon et al., 2016[79]). Moreover, a study conducted by Sun and colleagues in 2017 demonstrated that the use of the CRISPR/Cas9 system to knock out PSEN1 genes in N2a cells eliminated the background of endogenous γ-secretase. They also discovered that the introduction of recombinant protein derived from PSEN1 mutations decreased the production of Aβ42 and Aβ40 (Sun et al., 2017[92]).

A different research study on patient-derived fibroblasts revealed that the expression of Aβ protein declined when CRISPR/Cas9 technology was used to knock out Swedish APP (APPswe) mutations. This mutation, also known as Swedish KM670/671NL APP, causes an elevation in enzymatic cleavage via β-secretase, which in turn leads to increased levels of Aβ protein (György et al., 2018[31]). Additionally, a new mutation was introduced by Guyon et al. in 2021 through the use of a CRISPR/Cas9-based approach to modify the APP gene. They converted the alanine codon to threonine in both HEK293T cells and SH-SY5Y cells, which contain the APP gene with deaminated cytosine1 and cytosine2 positions. Their results revealed that the accumulation of Aβ peptide decreased further due to the successful introduction of the A673T mutation in 53 % of HEK293T cells, in addition to a novel mutation (E674K) (Guyon et al., 2021[30]).

The APOE (Apolipoprotein E) gene is presently acknowledged as a gene that makes an individual more susceptible to sporadic AD (SAD). APOE is predominantly produced by astrocytes in the central nervous system. APOE gene exists in different variants including APOE2 (ε2), APOE3(ε3), APOE3r, and APOE4 (ε4). The APOE4 form is the most potent genetic risk factor for SAD (Raulin et al., 2022[81]). In 2018, Lin and colleagues utilized hiPSC and CRISPR/Cas9 technology to determine the role of APOE4. Their findings indicated that APOE4 influenced Aβ metabolism in varying ways, depending on the specific cell type (Lin et al., 2018[51]). Furthermore, Wadhwani et al. in 2019 conducted research on potential therapeutic targets for APOE4. In their study, they used the CRISPR/Cas9 method to correct the E4 allele to the E3/E3 genotype in iPSCs from two individuals with AD. The results showed that E3 neurons were more resilient to ionomycin-induced cytotoxicity and had a reduction in tau phosphorylation compared to E4 neurons (Wadhwani et al., 2019[98]). 

### Application of CRISPR/Cas9 in Parkinson's disease

Parkinson's disease (PD) is the second most prevalent neurological disorder in humans, following Alzheimer's disease. It is a heterogeneous neurodegenerative condition identified by impaired bodily movements (Troncoso-Escudero et al., 2020[94]). PD is characterized by the progressive loss of dopaminergic neurons (DN) in the substantia nigra pars compacta (SNPC), which leads to a significant decrease in dopamine levels reaching the striatum and subsequent functional impairment of the motor circuit, resulting in motor symptoms like rest tremors, bradykinesia, and rigidity that constitute the core of its clinical features (Blesa et al., 2012[7]; Magrinelli et al., 2016[54]). Additionally, the presence of intracytoplasmic Lewy bodies (LB), primarily consisting of α-synuclein and ubiquitin, is also a defining characteristic. While α-synuclein gene mutations have only been linked to infrequent familial instances of PD, it is worth noting that α-synuclein is present in all Lewy bodies (Blesa et al., 2012[7]). Approximately 90 % of PD patients have no known cause (idiopathic), while the remaining 10 % have familial PD caused by mutations in specific genes like SNCA, PRKN/PARK2, PINK1, LRRK2, PARK7, DJ-1, GBA, UCH-L1, and MAPT/STH. It is possible that these mutations could also be associated with sporadic PD (Cota-Coronado et al., 2020[15]; Nalls et al., 2019[66]). The expression of α-synuclein is closely linked to SNCA gene, which is one of the most important predictive locations for sporadic PD (Ferreira and Massano, 2017[24]). The missense mutation called Ala53Thr (A53T) in SNCA is recognized as one of the most prominent risk factors for early-onset PD. SNCA has several mutations, but A53T is particularly noteworthy in its association with Parkinson's disease (Spira et al., 2001[91]). In 2022, Yoon et al. conducted a study which showed that using the CRISPR-Cas9 system to delete the A53T-SNCA gene significantly improved conditions related to Parkinson's disease, such as the overproduction of α-synuclein, reactive microgliosis, dopaminergic neurodegeneration, and motor symptoms associated with Parkinson's (Yoon et al., 2022[109]). 

In another study Kantor and colleges have developed a novel all-in-one lentiviral vector that employs CRISPR-Cas9 technology to specifically downregulation of SNCA mRNA and protein expression led to the reversal of disease-related phenotypic perturbations (Kantor et al., 2018[44]). Additionally, Chen and colleagues examined the mechanism by which SNCA operates in the nucleus using neurons derived from human-induced pluripotent stem cells from Parkinson's disease patients with A53T and SNCA-triplication autosomal dominant mutations, as well as their CRISPR-edited corrected counterparts. The study demonstrated that the absence of SNCA leads to resistance against Lewy pathology, indicating the possibility of utilizing CRISPR/Cas9n-mediated gene editing as a potential treatment for PD (Chen et al., 2020[12][13]). In another study Zhou et al. examined the PARK2 and PINK1 genes by utilizing CRISPR-Cas9 and somatic cell nuclear transfer techniques in a domestic pig model. The scientists revealed that they were able to acquire approximately 38 % successful outcomes in obtaining homozygous cell colonies that had a double-knock-out for PARK2 and PINK1 genes (Zhou et al., 2015[114]). Furthermore, a fascinating research conducted on nigral dopaminergic neurons (DN) involved the use of CRISPR/Cas system to delete PARKIN (PRKN), DJ-1 (PARK7), and ATP13A2 (PARK9) genes. By analyzing transcriptome and proteome data, it was found that oxidative stress is a shared dysregulation pathway among all isogenic cell lines (Ahfeldt et al., 2020[1]).

Loss-of-function mutations in DNAJC6, the gene responsible for producing HSP40 auxilin, have recently been found in individuals with early-onset PD. Human embryonic stem cells (hESCs) were used with CRISPR-Cas9-mediated gene editing to uncover these mutations. Through transcript analysis and experimental validation, it was discovered that defects in DNAJC6-mediated endocytosis can hinder the WNT-LMX1A signal during mDA neuron development. This, in turn, can lead to the generation of vulnerable mDA neurons with pathological symptoms as a result of reduced expression of LMX1A during development (Wulansari et al., 2021[102]).

### Application of CRISPR/Cas9 in Huntington's disease

Huntington's disease (HD) is a progressive neurodegenerative disorder caused by a single genetic mutation that follows an autosomal dominant pattern of inheritance. It is the most prevalent inherited neurodegenerative disease (Bates et al., 2015[5]). The mutation is characterized by the expansion of CAG (cytosine-guanine-adenine) repeats in the Huntingtin gene (HTT) (Paulsen, 2011[75]). This results in the formation of an elongated polyglutamine strand in the N-terminal region of the huntingtin protein (Kim et al., 2001[46]; Orr and Zoghbi, 2007[69]). This mutant protein aggregates in the brain, causing disruption to a wide variety of molecular and cellular processes (Waldvogel et al., 2015[99]), ultimately resulting in clinical symptoms such as progressive decline in cognitive function, chorea, dystonia, incoordination, and psychiatric disorders (Byun et al., 2022[10]). Because HD is caused by a single mutation and the presence of an abnormal protein, it is an appropriate candidate for gene therapy. CRISPR/Cas9 is one of the gene therapy approaches which used to suppress the expression of mutant HTT genes (Shin et al., 2016[87]).

Shin and colleagues conducted a study aimed at improving the specificity of alleles. They utilized a personalized allele-selective CRISPR/Cas9 strategy that was based on Protospacer Adjacent Motif (PAM)-altering SNPs. This strategy targeted CRISPR/ Cas9 sites specific to each patient and combined extensive knowledge of huntingtin (HTT) gene haplotype structure. The goal was to selectively inactivate the mutant HTT allele for a particular diplotype (Shin et al., 2016[87]). Additionally, Suzuki et al. developed a strategy called homology-independent targeted integration (HITI) that utilizes CRISPR/Cas9 technology to achieve efficient DNA knock-in in both dividing and non-dividing cells, both *in vitro *and* in vivo*. The effectiveness of HITI was shown in a rat model of retinitis pigmentosa, a condition that causes retinal degeneration, by improving visual function (Suzuki et al., 2016[93]). In another study, Yang et al. demonstrated that using CRISPR/Cas9 in HD140Q-KI mice to deplete HTT in a non-allele-specific manner can effectively and permanently eliminate polyglutamine expansion-induced neuronal toxicity in the adult brain. The experimental group exhibited a noteworthy decrease in reactive astrocytes and a marked improvement in motor dysfunction (Yang et al., 2017[108]). Furthermore, a study conducted by Ekman et al., which revealed that using CRISPR-Cas9 to disrupt the mutant HTT gene in R6/2 mice - that carry exon 1 of the human HTT gene with around 115-150 CAG repeats - resulted in a 2-fold reduction in the formation of neurotoxic inclusions. This also led to increased lifespan and improvement of specific motor deficits in the same mice, which highlights the potential of CRISPR-Cas9 technology as a treatment option for HD and reinforces its potential for addressing other autosomal dominant neurodegenerative disorders (Xie et al., 2019[103]). 

### Application of CRISPR/Cas9 in Amyotrophic lateral sclerosis

Amyotrophic lateral sclerosis (ALS), which is also referred to as “Lou Gehrig disease,” is a rapidly progressive neurodegenerative condition that impacts the human motor system. This condition is caused by the degeneration of motor neurons in the central nervous system (van den Bos et al., 2019[96]). ALS is the most prevalent type of motor neuron disease (MND), and it is identified clinically by dysfunction in both upper and lower motor neurons. This dysfunction leads to muscle weakness, atrophy, paralysis, and ultimately, respiratory failure and death (Oskarsson et al., 2018[71]). ALS can be categorized into two types: sporadic ALS (sALS), which accounts for about 90 % of cases and has an unknown inheritance pattern, and familial ALS (fALS), which constitutes approximately 10 % of all cases and is inherited (Boylan, 2015[8]). The most frequent pathogenic genes associated with ALS are C9orf72, SOD1, TARDBP, and FUS (Bursch et al., 2019[9]). Moreover, the hexanucleotide repeat expansion (HRE) in the noncoding area of the C9ORF72 gene has been identified as the most prevalent cause of both inherited (40 %) and sporadic (5-6 %) cases of ALS and FTD (Frontotemporal Dementia) (DeJesus-Hernandez et al., 2011[18]; Majounie et al., 2012[55]). In a study, Meijboom and colleagues utilized an adeno-associated viral vector system to transport CRISPR/Cas9 gene-editing machinery to remove HRE from the C9ORF72 genomic locus. This was successfully demonstrated in primary cortical neurons, mouse models, patient-derived iPSC motor neurons, and brain organoids. As a result, there was a decrease in RNA foci, poly-dipeptides, and haploinsufficiency, which are major indicators of C9-ALS/FTD. This is an encouraging therapeutic technique for treating these diseases (Meijboom et al., 2022[63]).

Deng et al. conducted a study where they used CRISPR/Cas9-mediated editing on transgenes (hSOD1-G93A) associated with ALS in two transgenic models. They showed that this *in vivo* gene-editing strategy is effective in targeting hSOD1, resulting in a disease-free state in two different hSOD1-G93A transgenic mouse models (G1H and G1L) of ALS (Deng et al., 2021[19]). Another study utilized the CRISPR/Cas9 method of gene correction to address the SOD1 E100G mutation. They performed targeted gene correction in induced pluripotent stem cells (iPSCs) obtained from a person with ALS linked to SOD1 and the E100G mutation. The iPSCs were later differentiated into motor neurons (Yun and Ha, 2020[110]). Furthermore, Chen et al. conducted a study wherein they outlined a CRISPR-Cas9 technique that is rapid, simple, and efficient for altering specific point mutations that are linked with ALS in human iPSCs, all without employing antibiotic selection. They rectified mutations like I114T in SOD1 in patient iPSCs or initiated mutations like G94A in SOD1 and H517Q in FUS in control iPSCs (Chen et al., 2021[11]).

### Application of CRISPR/Cas9 in Spinocerebellar ataxia

The spinocerebellar ataxias (SCAs) are a group of progressive neurodegenerative disorders primarily affecting the cerebellum that are inherited in an autosomal dominant manner (Soong and Morrison, 2018[90]). The primary clinical feature of SCAs is a gradual deterioration of balance and coordination, often accompanied by difficulties with speech. Symptoms usually appear during adulthood. SCAs are a heterogeneous group of disorders, with more than 40 genetically distinct subtypes identified to date. Each subtype is designated with the prefix SCA, followed by a sequential number that corresponds to the order in which the disease locus or the causative gene was discovered (Maas et al., 2015[53]). SCA1, SCA2, SCA3, and SCA6 are prevalent and clearly defined subtypes, comprising more than 50 % of cases, whereas the remaining cases consist of rare variants (Jacobi et al., 2018[35]). In genetic terms, the SCAs can be classified into two main categories: those that are a result of dynamic repeat expansion mutations and those caused by non-repeat mutations. Dynamic repeat expansions are also a significant factor in the occurrence of neurological diseases that are not categorized as SCAs (Maas et al., 2015[53]). There are at least 12 SCAs that are caused by repeat expansion mutations. Among these diseases, six of them - SCA1, SCA2, SCA3/Machado-Joseph disease (SCA3/MJD), SCA6, SCA7, and SCA17 - are specifically caused by translated CAG repeat expansion mutations that encode stretches of pure glutamine in the disease proteins. Because of this, these diseases are referred to as polyglutamine SCAs (Durr, 2010[20]; Paulson et al., 2017[76]).

In a study that employed CRISPR/Cas9 genome-editing therapy of SCA3, the primary objective of suppressing mATXN3 was effectively accomplished by eliminating the mutated CAG repeats in the ATXN3 gene through the CRISPR/Cas9 system, specifically by knocking out the mutant CAG expansions in exon 10. The NHEJ mechanism was predominantly responsible for repairing the gene, ultimately resulting in a truncated ataxin-3 protein that had a stop codon at the beginning of exon 11 (Ouyang et al., 2018[72]). Additionally, in a different study using the CRISPR/Cas9 genome editing technique of precise gene repair based on HR and paired sgRNAs, He et al. demonstrated that the use of paired sgRNAs/Cas9n and HR strategy can effectively repair the 74 CAG expansions in exon 10 of ATXN3 in SCA3-iPSCs (spinocerebellar ataxia type 3 patient-derived induced pluripotent stem cells), leading to a specific and efficient suppression of mutant ataxin-3 protein expression (He et al., 2021[32]). Moreover, by applying a CRISPR/Cas9 technology using homologous recombination, Song et al. established effective approaches for one-step genetic correction in SCA3-iPSCs of the SCA3 patients. Later, they advanced their research by developing SCA3 disease models in specific neurons differentiated according to the cerebellar region and disease-specific characteristics (Song et al., 2022[89]). A recent study has effectively created and confirmed a therapeutic plan using CRISPR/Cas9 for fibroblasts obtained from individuals with SCA1. The approach (using of G3 and G8 sgRNA/Cas9 complexes) efficiently decreased the production of both healthy and mutated ATXN1 protein (Pappadà et al., 2022[73]). These studies demonstrate that the precise gene repair approach of CRISPR/Cas9 genome editing is a promising treatment strategy for polyQ disorders. 

## Conclusion

In summary, the CRISPR/Cas9 genome editing technology holds great promise for the treatment of neurodegenerative diseases. The ability to selectively edit specific genes associated with these disorders presents a new avenue for developing targeted therapies. While still in its early stages, preclinical studies have shown encouraging results in animal models, demonstrating the potential for this technology to treat diseases such as Alzheimer's, Huntington's, Parkinson's, Amyotrophic lateral sclerosis, and Spinocerebellar ataxia. However, there are still many challenges that must be overcome before CRISPR/Cas9 can be translated to human therapies. Safety concerns, off-target effects, and delivery methods, all need to be carefully considered and optimized. Despite these challenges, the potential benefits of CRISPR/Cas9 for the treatment of neurodegenerative diseases cannot be ignored. It's essential to keep researching and developing this technology further if we want to unleash its maximum potential and offer hope to the millions of individuals who are suffering from these crippling disorders.

## Declaration

### Ethics approval and consent to participate

Not applicable.

### Consent for publication

Not applicable.

### Availability of data and materials

Not applicable.

### Competing interests

The authors declare that they have no competing interests.

### Funding

This research did not receive any specific grant from funding agencies in the public, commercial, or not-for-profit sectors. 

### Authors' contributions

During the research and manuscript preparation process, Jafar Nouri Nojadeh conducted preliminary research, conducted studies, classified and recorded available articles, and prepared initial manuscripts. Nur Seren Bildiren Eryılmaz contributed to the preparation of information and clinical characteristics of diseases. Berrin İmge Ergüder and Jafar Nouri Nojadeh collaborated on developing the overall concept of the review, finalizing the editing, and submitting the manuscript.

### Conflict of interest

The authors declare no conflict of interest.
